# Machine Learning-Based Prediction of Brain Tissue Infarction in Patients With Acute Ischemic Stroke Treated With Theophylline as an Add-On to Thrombolytic Therapy: A Randomized Clinical Trial Subgroup Analysis

**DOI:** 10.3389/fneur.2021.613029

**Published:** 2021-05-21

**Authors:** Boris Modrau, Anthony Winder, Niels Hjort, Martin Nygård Johansen, Grethe Andersen, Jens Fiehler, Henrik Vorum, Nils D. Forkert

**Affiliations:** ^1^Department of Neurology, Aalborg University Hospital, Aalborg, Denmark; ^2^Departments of Radiology & Clinical Neurosciences, University of Calgary, Calgary, AB, Canada; ^3^Department of Neurology, Aarhus University Hospital, Aarhus, Denmark; ^4^Unit of Clinical Biostatistics, Aalborg University Hospital, Aalborg, Denmark; ^5^Department of Neurology and Clinical Medicine, Aarhus University Hospital and Aarhus University, Aarhus, Denmark; ^6^Department of Diagnostic and Interventional Neuroradiology, University Medical Center Hamburg-Eppendorf, Hamburg, Germany; ^7^Department of Ophthalmology, Aalborg University Hospital, Aalborg, Denmark

**Keywords:** stroke, thrombolytic therapy, clinical trial, theophylline, reperfusion, machine learning, neuroprotective drugs

## Abstract

**Background and Purpose:** The theophylline in acute ischemic stroke trial investigated the neuroprotective effect of theophylline as an add-on to thrombolytic therapy in patients with acute ischemic stroke. The aim of this pre-planned subgroup analysis was to use predictive modeling to virtually test for differences in the follow-up lesion volumes.

**Materials and Methods:** A subgroup of 52 patients from the theophylline in acute ischemic stroke trial with multi-parametric MRI data acquired at baseline and at 24-h follow-up were analyzed. A machine learning model using voxel-by-voxel information from diffusion- and perfusion-weighted MRI and clinical parameters was used to predict the infarct volume for each individual patient and both treatment arms. After training of the two predictive models, two virtual lesion outcomes were available for each patient, one lesion predicted for theophylline treatment and one lesion predicted for placebo treatment.

**Results:** The mean predicted volume of follow-up lesions was 11.4 ml (standard deviation 18.7) for patients virtually treated with theophylline and 11.2 ml (standard deviation 17.3) for patients virtually treated with placebo (*p* = 0.86).

**Conclusions:** The predicted follow-up brain lesions for each patient were not significantly different for patients virtually treated with theophylline or placebo, as an add-on to thrombolytic therapy. Thus, this study confirmed the lack of neuroprotective effect of theophylline shown in the main clinical trial and is contrary to the results from preclinical stroke models.

## Introduction

The vasoactive agent theophylline has shown promising neuroprotective effects with reduced brain tissue edema, brain damage, and mortality in animal stroke models (1–3) but the results were controversial in previous randomized clinical trials (4, 5). The theophylline in acute ischemic stroke trial was designed to overcome the limitations of previous trials, namely the lack of acute ischemic stroke validation, lack of revascularization therapy, and delayed intervention (6). A total of 64 patients with acute ischemic stroke verified with MRI were randomized to a single infusion of 220 mg theophylline or placebo as an add-on to thrombolytic therapy. The co-primary endpoint of early clinical improvement, defined as change of the NIHSS score from baseline to follow-up at 24 h, improved by 4.7 points (standard deviation [SD] 5.6) in the theophylline group compared with an improvement of 1.3 points (SD 7.5) in the group treated with thrombolytic therapy alone (*p* = 0.04) (7). The co-primary endpoint of infarct growth at 24-h follow-up was 141.6% (SD 126.5) in the theophylline group and 104.1% (SD 62.5) in the control group (*p* = 0.15). While the clinical endpoint alone would have shown statistically significant early improvement, it was considered not statistically significant after correcting for multiple testing due to two primary endpoints. With respect to the imaging endpoint, comparing two independent groups with small sample size exhibiting a large variation of stroke lesion volumes might have prevented the detection of a small effect of theophylline. For that reason, a machine learning approach to predict follow-up lesions was pre-planned as a subgroup analysis. The basic idea of this method is to train two machine learning models based on imaging data on a voxel-wise basis acquired at the acute stage and the known follow-up lesion information. Thus, two predicted volumes of follow-up lesions can be quantified for each individual patient, one lesion outcome for the virtual treatment with theophylline and one lesion for the virtual treatment with placebo, which practically doubles the outcome measurements that can be used for statistical testing.

The aim of this study was to use this predictive modeling approach to compare follow-up lesion volumes of patients treated virtually with theophylline and placebo as an add-on to thrombolytic therapy to investigate if there is a subtle treatment effect of theophylline within individual patients that was not obvious when comparing the lesion volumes in the small groups.

## Materials and Methods

Anonymized data that support the findings of this study are available from the corresponding author upon reasonable request.

### Study Design

The present study is based on a subgroup of the theophylline in acute ischemic stroke trial, a current proof-of-concept, randomized, double-blinded, placebo-controlled trial that assessed the neuroprotective effect of theophylline as an add-on to thrombolytic therapy. The trial was registered at the European Union Drug Regulating Authorities Clinical Trials Database (EudraCT number 2013-001989-42). The trial protocol was approved by the Danish Health and Medicines Authorities (ref. no. 2013050908) and the Regional Scientific Ethic Committee (ref.-no. N-20130034) (6). Written informed consent was obtained from all patients and the trial was conducted in compliance with the Declaration of Helsinki. The trial was terminated early due to slow recruitment.

### Definition of Subgroup

The main selection criterion for the theophylline in acute ischemic stroke trial was eligibility for thrombolytic therapy within 4.5 h of symptom onset in patients with MRI verified moderate to severe acute ischemic stroke (NIHSS ≥4). For this pre-planned subgroup analysis, all patients with multi-parametric MRI including diffusion- and perfusion-weighted MRI at baseline, and available 24-h follow-up MRI were analyzed.

### Image Acquisition

All patients underwent MRI with the same field strength (1.5 or 3.0 Tesla) at admission (baseline) and at 24-h (22–32-h) follow-up. The MR sequences included diffusion-weighted MRI (DWI), perfusion-weighted MRI (PWI) with intravenous gadolinium (0.1 mmol per kilogram body weight, 5 ml/s bolus injection), circle of Willis time-of-flight MR angiography, gradient echo weighted MRI, and T2-FLAIR MRI. The thrombolysis in myocardial infarction (TIMI) grading was used to grade arterial obstruction (8). TIMI 0-1 at baseline was defined as large vessel occlusion and TIMI 0-1 at baseline converted to 3-4 at 24-h follow-up was defined as revascularization.

### Image Post-processing

Post-processing of DWI and PWI was performed semi-automatically using the AnToNIa software tool to extract voxel-by-voxel information for training of the machine learning models (9). In detail, the following processing steps were applied: An apparent diffusion coefficient dataset was calculated based on two DWI datasets (*b*-value = 0 and *b*-value,= 1,000 mm^2^/s). The ADC map was used for an automatic thresholding-based brain tissue and cerebrospinal fluid segmentation. The baseline ischemic core was manually delineated using a semi-automatic volume growing approach with an upper ADC threshold of 550 × 10^−6^ mm^2^/s, in accordance to a previous stroke study (10). The delineated ischemic core was then used to compute a distance map displaying the Euclidean distance for each non-core voxel to the closest voxel of the ischemic core. This was done to incorporate prior knowledge that the probability of final tissue infarction decreases with distance to the infarct core into the machine learning model. The PWI dataset was automatically motion corrected and the arterial input function was manually defined at the ICA or MCA M1-segment. A block-circulant deconvolution-based perfusion analysis was used to calculate the cerebral blood flow, cerebral blood volume, mean transit time, and time-to-maximum of the residual function (Tmax). Mean values were determined for each parameter in the contralateral brain tissue (excluding cerebrospinal fluid) and used for normalization of the perfusion parameters (CBF and CBV values by calculating the ratio, MTT and Tmax by subtraction of the contralateral average values). The normalized Tmax perfusion parameter maps were segmented using a lower threshold of 6 s to determine the hypoperfused tissue. The PWI parameter maps were automatically registered to the ADC dataset and the tissue at risk was calculated by subtracting the ADC lesion from the Tmax (>6 s) hypoperfusion. Information about the localization of each voxel and possible regional vulnerability to ischemia was added by registering the Montreal Neurological Institute brain atlas to each ADC dataset, described in more details by Forkert et al. (9). The ipsilateral follow-up brain tissue lesion was manually segmented in the follow-up T2-FLAIR dataset of each patient acquired at 24-h using the software tool ITK-SNAP (v. 3.6.0) (11) and registered to the baseline ADC dataset. The imaging data were complemented by the following clinical parameters: age, sex, baseline NIHSS, and time of stroke onset to theophylline / placebo application.

Finally, 12 equally weighted features were available for each voxel: Tissue type probability, anatomical location, distance to the ischemic core, ADC value, CBF value, CBV value, MTT value, Tmax value, and the four clinical parameters as well as information about the infarct outcome. A stratified, under-sampled training set consisting of all voxels from the follow-up infarct lesion and an equal number of non-lesion voxels randomly sampled from the ipsilateral hemisphere were extracted for each dataset. This approach and feature setup yielded the best results in a previous in-depth technical evaluation (12).

### Lesion Outcome Prediction

Two random forest machine learning models were trained, one using all training sets from the patients treated with theophylline and one using all training sets from the patients treated with placebo. The patient in whom the algorithm was tested (used for lesion outcome prediction) in each iteration was always excluded from the aggregated training set used for training of the random forest models. Practically, the machine learning models were implemented using ALGLIB (www.alglib.net) (13) with the random forest model consisting of 100 trees assuring an acceptable compromise between accuracy and computation speed, described in more details by Winder et al. (12).

After training of the two predictive models, both models were used to predict the lesion outcome for both treatment options for each patient.

### Statistical Analyses

Baseline characteristics are presented by the mean ± standard deviation or median and interquartile range, as appropriate. Continuous data were compared with Wilcoxon's rank sum test. Categorical data were summarized by counts and percentages and compared using Fisher's exact test. For the primary endpoint, a paired *t*-test was used to compare the predicted volumes of the follow-up lesion for each patient virtually treated with theophylline or placebo and the data was represented as box-whisker graph. Dice similarity coefficients comparing the true lesion outcome with the predicted lesion outcome within each group were calculated to quantify the prediction accuracy. Furthermore, the difference and average of both predictions for each patient were calculated and presented using a Bland Altman plot. For the additional explorative analysis, an unpaired *t*-test was used to compare the difference between the two predicted lesion volumes for patients with and without presence of tissue at risk at baseline, for patients with cortical vs. lacunar infarction, for patients with large vessel occlusion at baseline, and for patients with recanalization at follow-up.

All tests were applied as *post-hoc* analysis with a two-sided alpha level of 0.05 without correction for multiple comparisons. Stata/MP version 15.1 (Stata Corp LLC) was applied for the analysis.

## Results

### Study Population

MRI including baseline DWI and PWI and follow-up T2-FLAIR at 24 h was available in 52 out of 64 patients included in the main study (43 patients imaged at 3.0 Tesla and 9 patients imaged at 1.5 Tesla MRI field strength). Overall, the baseline characteristics were similar between the two treatment groups (Table 1). However, diabetes mellitus was more frequently present in the control group (four patients) compared to the theophylline group (no patients). The process measures: time of stroke onset to door, door to thrombolysis, door to theophylline/placebo treatment, and the rate of additional endovascular therapy were similar between the two treatment groups. Likewise, the baseline imaging characteristics volume of ischemic core, volume of tissue-at-risk, type of vessel occlusion, and grade of vessel occlusion were similar between the two treatment groups. The mean volume of ischemic lesion based on the segmentation of the follow-up T2-FLAIR dataset at 24-h was 13.9 ml (SD 20.3) for the theophylline group and 11.7 ml (SD 19.3) for the placebo group (*p* = 0.92).

**Table 1:** Baseline characteristics.

	**Theophylline group (*N* = 27)**	**Control group (*N* = 25)**	***p*-value**
**Clinical**			
Median age – year (IQR)	71 (55–77)	69 (52–78)	0.86
Female sex – no. (%)	10 (37)	11 (44)	0.61
Median NIHSS score (IQR)	8 (6–13)	7 (6–8)	0.12
Hypertension – no. (%)	14 (52)	16 (64)	0.38
Diabetes mellitus – no. (%)	0 (0)	4 (8)	0.03
Hyperlipidemia – no. (%)	16 (59)	16 (64)	0.73
Arterial fibrillation – no. (%)	2 (7)	4 (16)	0.33
Peripheral arterial disease – no. (%)	2 (7)	0 (0)	0.16
Previous myocardial infarction – no. (%)	1 (4)	1 (4)	0.96
Previous transitory ischemic attack – no. (%)	2 (7)	2 (8)	0.94
Previous stroke – no. (%)	3 (11)	3 (12)	0.92
Previous intracranial hemorrhage – no. (%)	0 (0)	0 (0)	1
Current smoking – no. (%)	10 (37)	9 (36)	0.94
Antiplatelet agent – no. (%)	7 (26)	9 (36)	0.43
**Imaging characteristics**			
Mean volume of ischemic core – ml (SD)	4.4 (6.4)	4.3 (7.8)	0.81
Mean volume of baseline tissue at risk – ml (SD)	9.6 (22.0)	6.5 (13.9)	0.44
Large vessel occlusion – no. (%)	12 (44)	11 (44)	0.97
**Type of vessel occlusion**			0.85
Anterior cerebral artery – no. (%)	0 (0)	1 (4)	
Middle cerebral artery (M1-segment) – no. (%)	6 (22)	4 (16)	
Middle cerebral artery (M2-3-segment) – no. (%)	5 (19)	5 (20)	
Posterior cerebral artery – no. (%)	1 (4)	1 (4)	
No visible large vessel occlusion – no. (%)	15 (55)	14 (56)	
**Grade of vessel occlusion at baseline**			0.97
TIMI-score 0–1 – no. (%)	12 (44)	11 (44)	
TIMI-score 2–3 – no. (%)	15 (56)	14 (56)	
**Process measures**			
Median stroke onset – door time – min (IQR)	81 (48–148)	85 (68–130)	0.41
Median door to thrombolysis-time – min (IQR)	39 (35–45)	41 (35–51)	0.75
Additional endovascular therapy – no. (%)	3 (11)	4 (16)	0.61

*The baseline characteristics of the two groups were not significantly different apart from diabetes mellitus (p = 0.03)*.

*IQR, Interquartile range; SD, Standard deviation; NIHSS, National Institute of Health Stroke Scale; TIMI, thrombolysis in myocardial infarction grading of arterial obstruction (score zero, complete occlusion; one, severe stenosis; two, mild to moderate stenosis; three, normal arterial caliber)*.

### Primary Outcome

The mean Dice similarity coefficient was 0.40 (SD 0.249) for the theophylline prediction model and 0.35 (SD 0.243) for the placebo prediction model, which is in the range of previously described methods (12).

**Figure 1:**
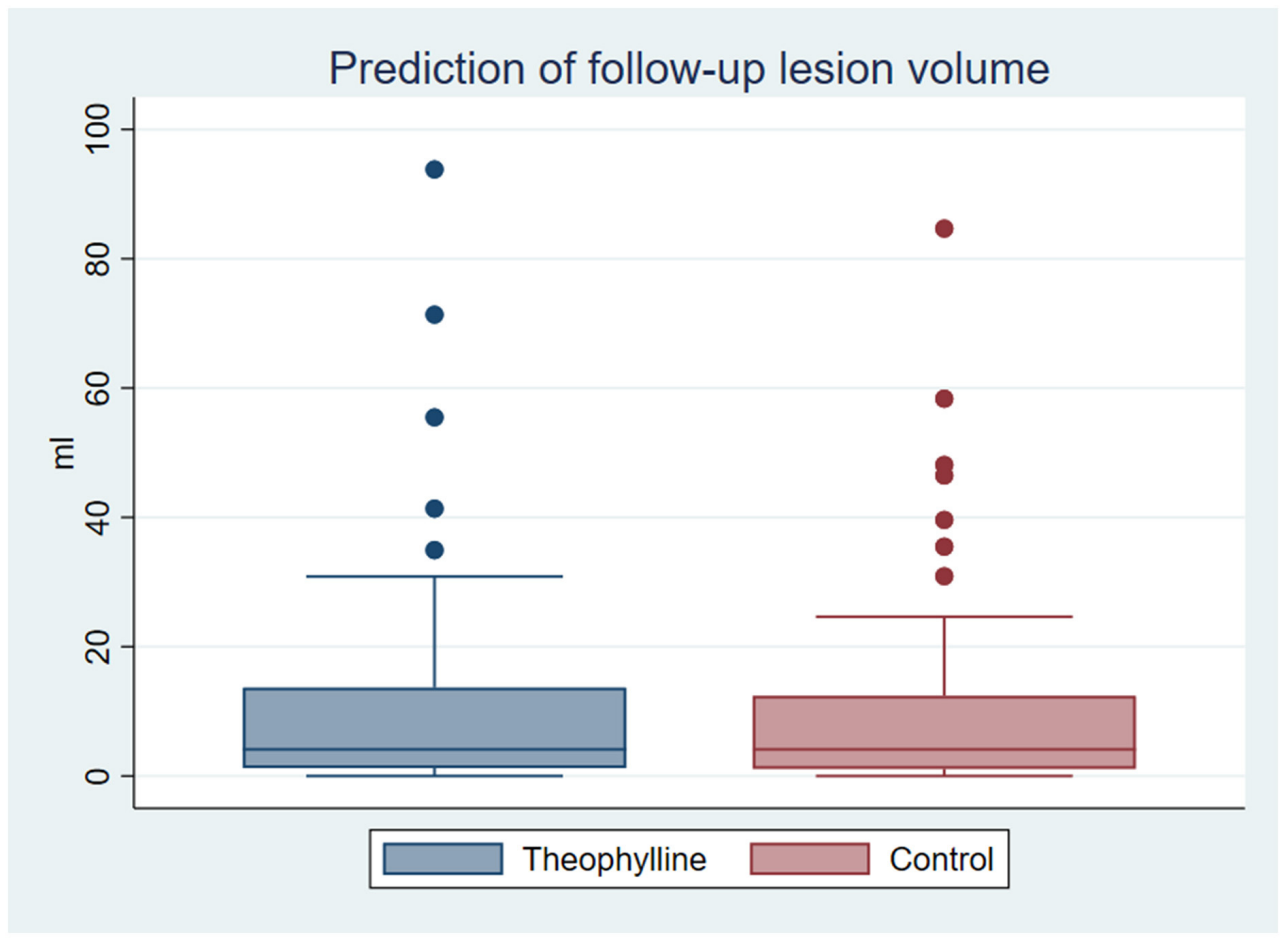
Predicted volume of follow-up lesions. Box-whisker diagram depicting the mean predicted volumes of follow-up lesions based on the trainings datasets of patients treated with theophylline and placebo: Interquartile range with median (box), lower and upper interquartile +1.5 interquartile range (whisker), and outliers (dots).

Using the two predictive models, the mean predicted lesion volume was 11.4 ml (standard deviation (SD) 18.7) for the patients virtually treated with theophylline and 11.2 ml (SD 17.3) for patients virtually treated with placebo (Figure 1). No significant difference was found between the predicted volumes of the two treatment groups using a paired *t*-test (*p* = 0.86). A detailed list of the true and predicted lesion volumes for each individual patient is provided as Supplementary Table 1.

### Additional Analyses

**Figure 2:**
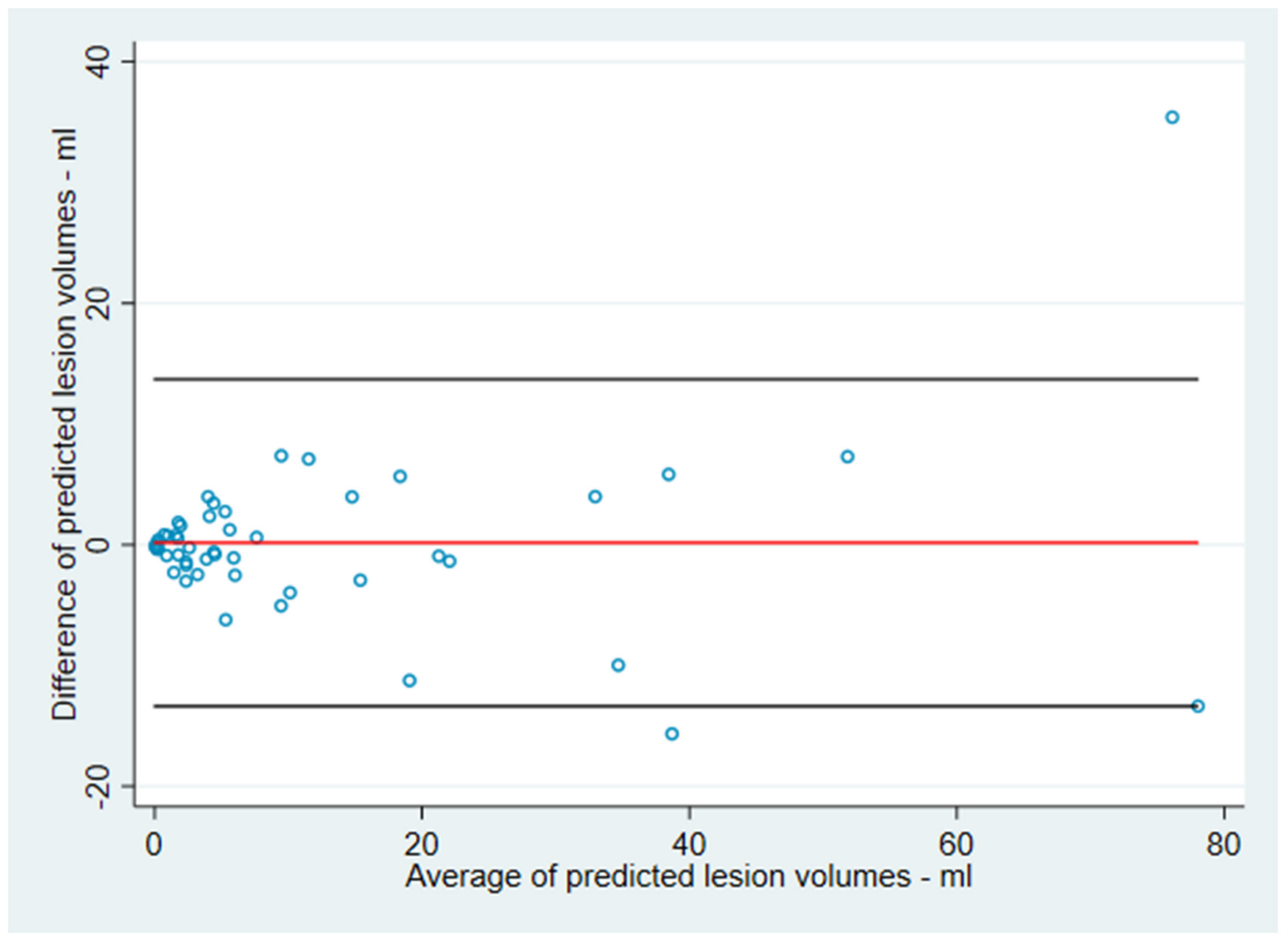
Interaction of infarct size and prediction. Bland Altman plot depicting the predicted volume of follow-up lesions plotted as the difference between the two predictions (theophylline and placebo) over the average of the two predicted volumes for each patient: Values <0 indicate that theophylline is better than placebo while values >0 indicate that placebo is better than theophylline: Middle line indicates mean difference between the predicted values, upper and lower line indicates the higher and lower limit of agreement (2x standard deviation) indicating no significant difference between small and large infarct volumes.

**Figure 3:**
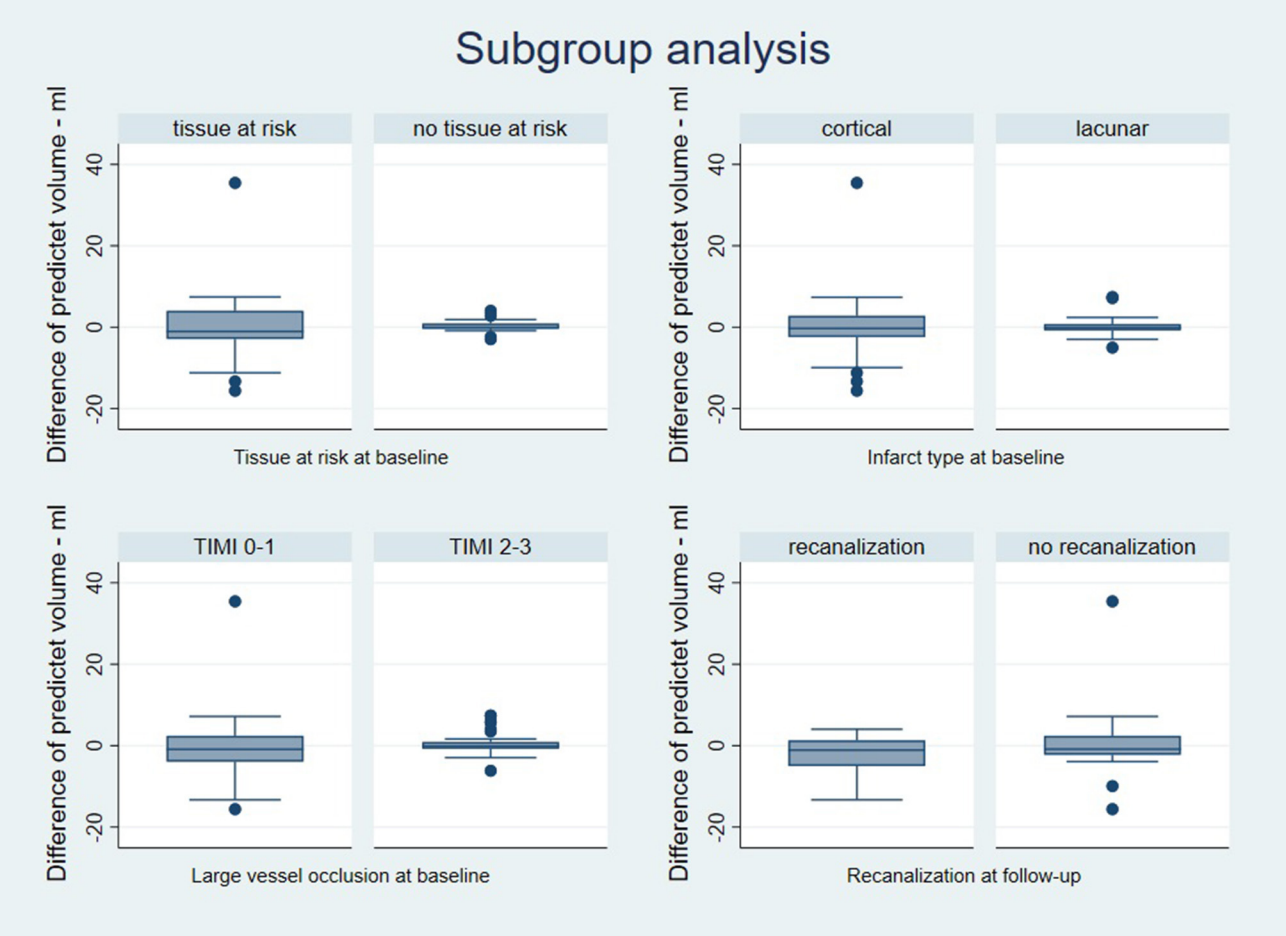
Subgroup analysis. Box-whisker diagrams depicting the predicted volume of follow-up lesions plotted as the difference between the two predictions (theophylline and placebo) for each patient: Values <0 indicate that theophylline is better than placebo and values >0 indicate that placebo is better than theophylline indicating no significant difference for patients with and without tissue-at-risk at baseline, for patients with cortical vs. lacunar infarction, for patients with and without large vessel occlusion at baseline, and for patients with and without recanalization at follow-up.

The predicted lesion volumes for each patient virtually treated with theophylline or placebo were similar for small and large infarct volumes as illustrated in the Bland-Altman plot (Figure 2). Tissue-at-risk at baseline was present in 29 patients and absent in 23 patients. The type of stroke was classified as cortical in 34 patients and lacunar in 18 patients. Twenty-three patients fulfilled the criteria for large vessel occlusion at baseline (TIMI 0-1), and the criteria for recanalization at follow-up (TIMI 3-4) were fulfilled in 10 out of these 23 patients. The predicted follow-up lesions were not significantly different between patients with and without presence of tissue-at-risk at baseline (*p* = 0.89), in patients with cortical vs. lacunar infarction (*p* = 0.88), in patients with and without large vessel occlusion at baseline (*p* = 0.57), and in patients with and without recanalization at follow-up (*p* = 0.35) (Figure 3).

## Discussion

The main finding of this secondary analysis of the theophylline in acute ischemic stroke trial is that the predicted follow-up lesion volumes of patients virtually treated with theophylline or placebo are not significantly different.

The primary aim of the theophylline in acute ischemic stroke trial was to investigate the neuroprotective effect of theophylline as an add-on to thrombolytic therapy in patients with acute ischemic stroke. The clinical endpoint alone would have shown a statistically significant early improvement at 24-h follow-up (*p* = 0.04) but was considered not statistically significant after correcting for multiple testing due to two primary endpoints. The co-primary endpoint infarct growth did not reach significance (*p* = 0.19). However, it was uncertain whether the considerable variation of stroke lesion volumes in a small sample size and the infarct growth information limited to the follow-up T2-FLAIR segmentation might have prevented detection of a subtle effect of theophylline. The rather small infarct volumes and the large variation in a small sample size was the main motivation for this *post-hoc* analysis using a predictive modeling approach for a more in-depth comparison of the groups. Various machine learning models have been presented in the past for this purpose typically showing better performance for tissue outcome prediction compared to simple perfusion parameter thresholding (14–17). A recent study showed that such machine learning models cannot only be used to predict the lesion outcome in patients for treatment decision making but also for intra-individual virtual comparisons of treatments (18). This voxel-by-voxel analysis of eight imaging parameters for each voxel based on DWI and PWI sequences collected from each patient together with four clinical parameters practically allows to double the sample size by simulating both treatment outcomes for each patient included in this study. The accuracy of the prediction models was acceptable as the mean Dice similarity coefficients comparing the true lesion volumes with the predicted lesion volumes within each group were within the range of results from a recent study applying multi-parametric tissue outcome prediction methods (12).

In our trial, the surrogate marker for follow-up lesion volume was almost identical for patients virtually treated with theophylline and patients virtually treated with placebo. Thus, the machine learning approach with practically doubled sample size of outcome measurements compared to the main clinical trial confirmed the lack of a neuroprotective effect of theophylline. This is most notably true, as there is no detectible neuroprotective effect on infarct growth in the highly selected subgroup of patients with presence of tissue-at-risk at baseline and/or subsequent recanalization. One limitation of this study is that the small sample size did not allow a sufficient investigation of a lesion-size dependent effect of theophylline although the Bland Altman plots as well as a simple evaluation using regression analysis do not suggest such an effect. Although all perfusion MRI were sufficient for analysis, and complete follow-up was available, the main limitation remains the low number of patients in this subgroup analysis. However, previous work has shown that the machine learning model used requires no more than 50 datasets for optimal training (19). Accordingly, the Dice scores achieved in our study were within the range of previously published papers. Another limitation is that machine learning models are rarely employed in clinical trials. As discussed by Fiehler et al. (18), the potential of a study design with virtual comparators based on predictive modeling is highly informative, quick, and relatively inexpensive. In contrast to that, the established study design of a randomized control trial provides the highest level of evidence. In our study, application of a machine learning model allowed further insights given the limited dataset and confirmed the results of the main trial. However, the large variation of lesions, the variation of infarct types with and without collateral supply, and the large variation or lack of tissue-at-risk are still relevant limitations of this study. Nevertheless, we believe that predictive modeling to virtually test for differences in the follow-up lesion volumes can become an essential tool to support conventional trials in acute ischemic stroke by enabling an improved evaluation of evidence on intermediate outcomes and to increase the sample size for the primary or secondary outcomes in case of underpowered studies.

There is no known interaction between theophylline and alteplase. Recently, such interaction was observed to inhibit the neuroprotective effect of Nerinetide in acute ischemic stroke in the ESCAPE-NA1-trial. In that trial, Nerinetide did not improve the good clinical outcomes after endovascular thrombectomy (20).

In line with numerous previous neuroprotective clinical trials, our trial failed to translate the promising results of reduced brain infarction from pre-clinical stroke models to humans (21). However, from a patient and physician perspective, neuroprotection means keeping the damage of the ischemic stroke below the threshold of symptom manifestation (22). For that reason, a lack of an effect on the surrogate marker infarct growth does not outrange the clinical response and the neuroprotective effect of theophylline should still be investigated in more detail.

In summary, predictive modeling using advanced machine learning was performed to uncover potential subtle effects on follow-up lesion volumes of theophylline as an add-on to thrombolytic therapy in patients with acute ischemic stroke. The predicted follow-up brain lesions for each patient virtually treated with theophylline and placebo confirmed the volumetric results based on the small sample sizes of the original study. Thus, this study also confirmed the lack of neuroprotective effect of theophylline shown in the main clinical trial and is contrary to the results from preclinical stroke models.

## Data Availability Statement

The raw data supporting the conclusions of this article will be made available by the authors, without undue reservation.

## Ethics Statement

The studies involving human participants were reviewed and approved by Danish Regional Scientific Ethic Committee (ref.-no. N-20130034). The patients/participants provided their written informed consent to participate in this study.

## Author Contributions

BM, AW, NH, JF, and NF contributed to the conception and design of the study. BM, NH, AW, and NF contributed to the acquisition and analysis of the data. BM wrote the main draft. All authors discussed the results and implications and contributed to the interpretation and critical revision of the data. BM and NF finalized and submitted the manuscript.

## Conflict of Interest

The authors declare that the research was conducted in the absence of any commercial or financial relationships that could be construed as a potential conflict of interest.
