# Supplementary material for: Machine Learning-Based Prediction of Brain Tissue Infarction in Patients With Acute Ischemic Stroke Treated With Theophylline as an Add-On to Thrombolytic Therapy: A Randomized Clinical Trial Subgroup Analysis
Source: Front Neurol. 2021 May 21;12:613029. doi: 10.3389/fneur.2021.613029 (PMC8175622; doi:10.3389/fneur.2021.613029)
Supplement: Supplementary file 1 [file Table_1.docx]

# Supplementary material

**Supplementary Table 1. True and predicted follow-up lesion volumes for each individual patient**

| Active | Follow-up lesion volume (ml) | | |  | Placebo | Follow-up lesion volume (ml) | | |
| --- | --- | --- | --- | --- | --- | --- | --- | --- |
| Subject | **True** | **Predicted Active** | **Predicted Placebo** |  | **Subject** | **True** | **Predicted Active** | **Predicted Placebo** |
| 2 | 41,8 | 34,9 | 30,9 |  | **5** | 7,8 | 5,4 | 6,4 |
| 3 | 7,7 | 6,9 | 11,9 |  | **6** | 38,3 | 21,4 | 22,7 |
| 4 | 49,0 | 16,7 | 12,7 |  | **8** | 5,8 | 2,7 | 0,8 |
| 7 | 13,2 | 0,0 | 0,3 |  | **9** | 42,8 | 21,2 | 15,5 |
| 10 | 31,6 | 55,5 | 48,1 |  | **11** | 3,1 | 4,1 | 4,9 |
| 12 | 31,9 | 41,3 | 35,5 |  | **13** | 6,2 | 4,1 | 4,7 |
| 14 | 1,7 | 6,2 | 4,9 |  | **15** | 2,6 | 6,6 | 3,8 |
| 16 | 2,1 | 4,7 | 7,2 |  | **18** | 1,7 | 2,0 | 1,1 |
| 17 | 75,2 | 71,3 | 84,7 |  | **22** | 4,0 | 2,2 | 8,4 |
| 19 | 4,0 | 1,3 | 2,1 |  | **23** | 26,4 | 6,1 | 2,6 |
| 20 | 57,1 | 20,8 | 21,7 |  | **24** | 10,9 | 13,4 | 24,6 |
| 27 | 11,4 | 2,0 | 4,4 |  | **25** | 18,8 | 29,7 | 39,6 |
| 32 | 0,5 | 0,1 | 0,4 |  | **28** | 15,0 | 8,1 | 12,0 |
| 34 | 5,6 | 2,5 | 2,6 |  | **29** | 1,5 | 2,7 | 1,1 |
| 35 | 1,3 | 2,0 | 1,4 |  | **33** | 85,2 | 93,8 | 58,3 |
| 36 | 4,4 | 5,3 | 2,9 |  | **40** | 1,6 | 1,6 | 3,0 |
| 39 | 9,4 | 13,1 | 5,7 |  | **45** | 0,7 | 0,8 | 3,8 |
| 42 | 0,0 | 0,0 | 0,3 |  | **47** | 13,1 | 13,9 | 16,8 |
| 46 | 0,6 | 1,1 | 0,2 |  | **49** | 0,2 | 0,0 | 0,2 |
| 50 | 0,1 | 0,0 | 0,0 |  | **53** | 0,6 | 0,0 | 0,2 |
| 51 | 2,7 | 7,9 | 7,3 |  | **55** | 0,9 | 0,0 | 0,2 |
| 56 | 1,0 | 1,4 | 0,6 |  | **57** | 2,0 | 3,3 | 4,4 |
| 60 | 0,6 | 0,3 | 0,1 |  | **59** | 0,4 | 6,0 | 1,9 |
| 62 | 0,4 | 0,5 | 0,0 |  | **61** | 0,6 | 0,4 | 1,3 |
| 66 | 15,6 | 15,1 | 7,9 |  | **63** | 0,2 | 1,5 | 3,1 |
| 67 | 2,5 | 0,3 | 2,5 |  | **65** | 3,9 | 30,9 | 46,5 |

Left block: Patients treated with theophylline. Right block: Patients treated with placebo. The true follow-up brain tissue lesion was manually segmented in the follow-up T2-FLAIR dataset acquired at 24-hours in the 52 patients with available perfusion imaging. After training of the two predictive models, both models were used to predict the lesion outcome for the active and placebo treatment option for each patient.
